# A coumarin–dihydroperimidine dye as a fluorescent chemosensor for hypochlorite in 99% water†

**DOI:** 10.1039/c9ra05533a

**Published:** 2019-09-11

**Authors:** Yasuhiro Shiraishi, Chiharu Yamada, Takayuki Hirai

**Affiliations:** Research Center for Solar Energy Chemistry, Division of Chemical Engineering, Graduate School of Engineering Science, Osaka University Toyonaka 560-8531 Japan shiraish@cheng.es.osaka-u.ac.jp

## Abstract

The hypochlorite anion (OCl^−^), a reactive oxygen species (ROS), is an important microbicidal agent in the immune system. Accurate and selective detection of OCl^−^ in environmental and biological samples by a fluorescent molecular sensor is an important subject. All previously reported sensors, however, have suffered from tedious multi-step synthesis for the sensors and the use of large amounts of organic solvents for the analysis. Herein, we report that a coumarin–dihydroperimidine dye prepared by facile condensation behaves as a fluorescent sensor for OCl^−^ in 99% water. The sensor exhibits weak fluorescence, but OCl^−^-selective dehydrogenation of its dihydroperimidine unit creates a strong blue fluorescence. This turn-on fluorescence response facilitates selective and sensitive detection of OCl^−^ in the physiological pH range. *Ab initio* calculation revealed that the fluorescence enhancement by OCl^−^ is triggered by intramolecular proton transfer from the coumarin –OH to the imine nitrogen of the formed perimidine moiety.

## Abstract 2

A coumarin–dihydroperimidine dye exhibits strong blue fluorescence by OCl^−^-selective dehydrogenation of the dihydroperimidine unit, and facilitates selective and sensitive fluorometric detection of OCl^−^ in 99% water.

## Graphical abstract

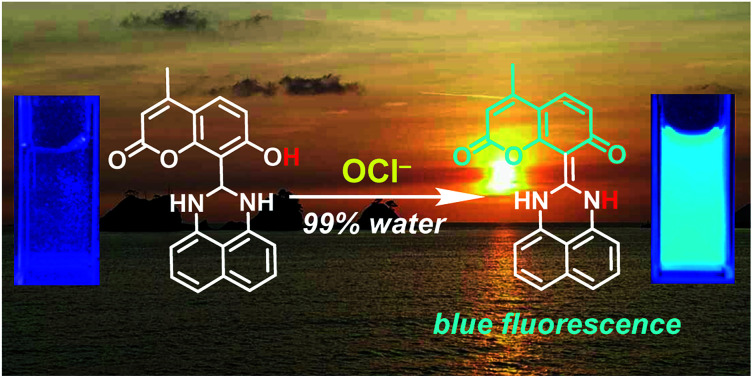

## Introduction

Reactive oxygen species (ROS) play crucial roles in several life functions.^1^ Among them, hypochlorous acid (HClO) is one of the most biologically important ROS.^2^ HClO undergoes deprotonation at physiological pH and produces the hypochlorite anion (OCl^−^),^3^ which behaves as a microbicidal agent in the immune system.^4^ OCl^−^ is produced *in vivo* by the reaction of hydrogen peroxide (H_2_O_2_) with Cl^−^*via* an enzymatic reaction on myeloperoxidase (MPO).^5^ Controlled generation of OCl^−^ is necessary to inhibit invading microbes. Uncontrolled OCl^−^ generation, however, causes several diseases such as neuron degeneration, arthritis, and cancer,^6^ because OCl^−^ reacts with several biomolecules such as amino acids, proteins, and nucleosides.^7^ In addition, HClO is widely used in daily life for sterilization and disinfection of water supplies, and high residual concentrations of OCl^−^ in water is hazardous to human and animal health.^8^ Analytical methods that quantitatively detect small amount of OCl^−^ in environmental and biological samples on inexpensive instrumentations with simple pre-treatment are necessary.

**Scheme 1:**
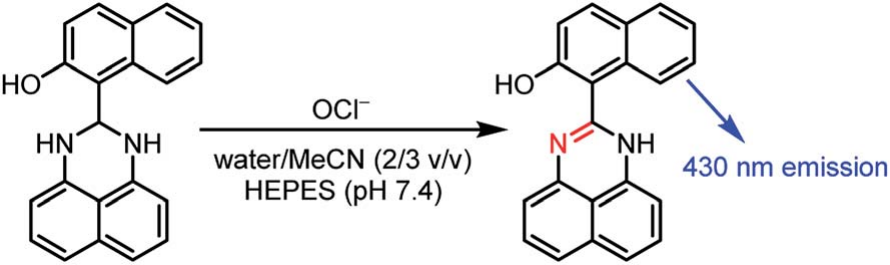
A naphthol–dihydroperimidine dye exhibiting a turn-on fluorescence response toward OCl^−^.^18^

Fluorometric analysis with OCl^−^-selective molecular sensors is one promising method for this purpose since this facilitates simple quantification or imaging of OCl^−^ with a common fluorescence spectrometer or microscope apparatus.^9^ A number of fluorescent OCl^−^ sensors have been reported;^10–17^ however, many of them require tedious multi-step procedures for the synthesis of sensors or a solution containing a large amount of organic solvents for sensing due to the low solubility of the sensors in water. Among the previously reported OCl^−^ sensors, a “dihydroperimidine”-based sensor designed by Goswami *et al.*^18^ has the simplest structure, which can be prepared by a facile condensation. As shown in Scheme 1, they synthesized a naphthol–dihydroperimidine dye by the condensation of 1,8-diaminonaphthalene with 1-formyl-2-naphthol as a fluorophore. The sensor shows a sensitive turn-on fluorescence response *via* an OCl^−^-selective dehydrogenation of the dihydroperimidine unit. The sensor, however, requires a solution containing 60% MeCN owing to its low solubility in water. Based on this molecular design, Fan *et al.*^19^ synthesized a sensor by the condensation of 1,8-diaminonaphthalene with 7-diethylamino-1,4-benzoxazin-2-one as a fluorophore. Although the sensor exhibits a selective and sensitive response towards OCl^−^, it still requires a large amount of organic solvent (80% DMF) for the sensing. Design of a sensor that can be synthesized by a simple procedure and has a high water solubility is therefore desirable.

**Scheme 2:**
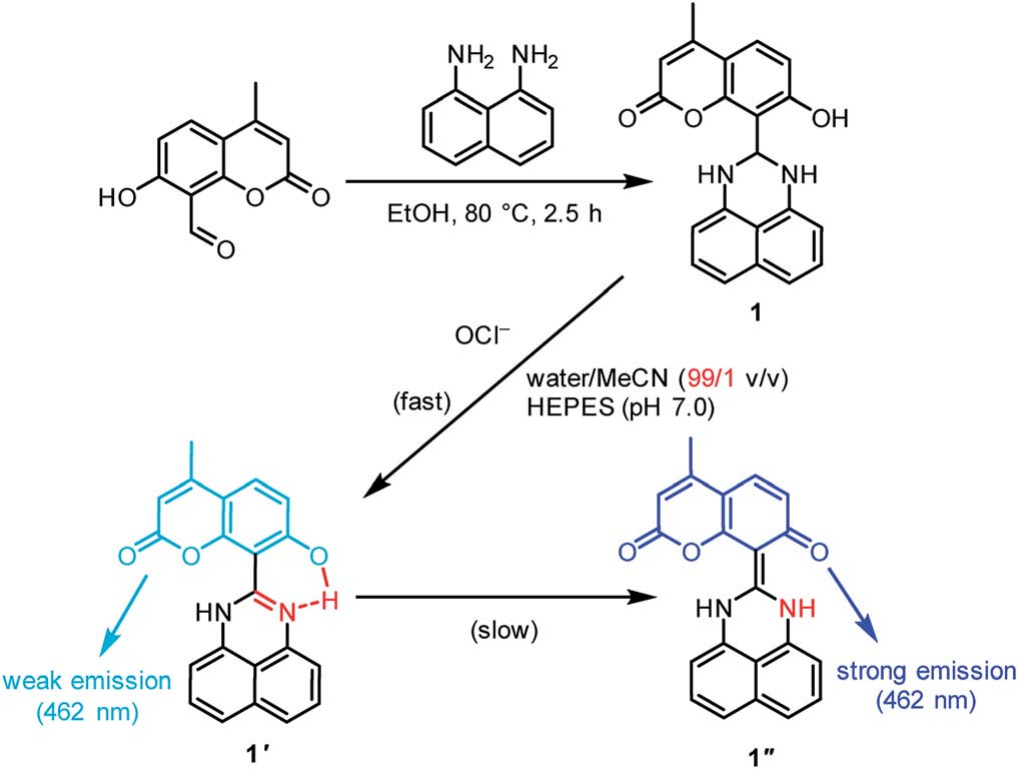
Synthesis of the sensor 1, and proposed mechanism for selective turn-on fluorescence response by OCl^−^.

We used coumarin as a fluorophore due to its relatively high water solubility,^20^ high fluorescence quantum yield,^21^ large Stokes shift,^22^ high stability,^23^ and good cell permeability.^24^ As shown in Scheme 2, the sensor 1, synthesized by a simple condensation of 1,8-diaminonaphthalene with 8-formyl-7-hydroxy-4-methylcoumarin, is soluble in water containing only 1% organic solvents. The sensor shows a weak fluorescence, but OCl^−^-selective dehydrogenation of its dihydroperimidine unit creates a strong fluorescence at 462 nm. This turn-on response facilitates sensitive detection of OCl^−^. Several spectroscopic analysis and *ab initio* calculations revealed that this turn-on response by OCl^−^ is triggered by intramolecular proton transfer from the coumarin –OH to the imine nitrogen of the formed perimidine unit.

## Results and discussion

### Synthesis and fluorescence properties of the sensor

The sensor 1 was prepared by the reaction shown in Scheme 2. 8-Formyl-7-hydroxy-4-methylcoumarin prepared by formylation of 7-hydroxy-4-methylcoumarin (yield: 45%)^25^ and 1,8-diaminonaphthalene were dissolved in EtOH, and the solution was stirred at 80 °C for 2.5 h in an aerated condition. The solid formed was recovered by filtration and washed thoroughly with EtOH, affording 1 as pale pink solids with 69% yield (overall yield: 31%). The purity of 1 was confirmed by ^1^H NMR, ^13^C NMR and FAB-MS analysis (Fig. S1–S3, ESI†). 1 is soluble in common organic solvents such as DMSO, CHCl_3_, DMF, and MeCN and in aqueous solutions with 1% organic solvents such as DMSO and MeCN. Fig. S4 (ESI†) shows the absorption spectra of 1% MeCN solutions containing different concentrations of 1. The linear relationship between the absorbance at 325 nm and the concentration of 1 (0–20 μM) indicates that it follows the Beer's law, suggesting that 1 is fully soluble in the solutions. Note that the molar extinction coefficient of 1 at 325 nm was determined to be 10 039 M^−1^ cm^−1^.

**Fig. 1:**
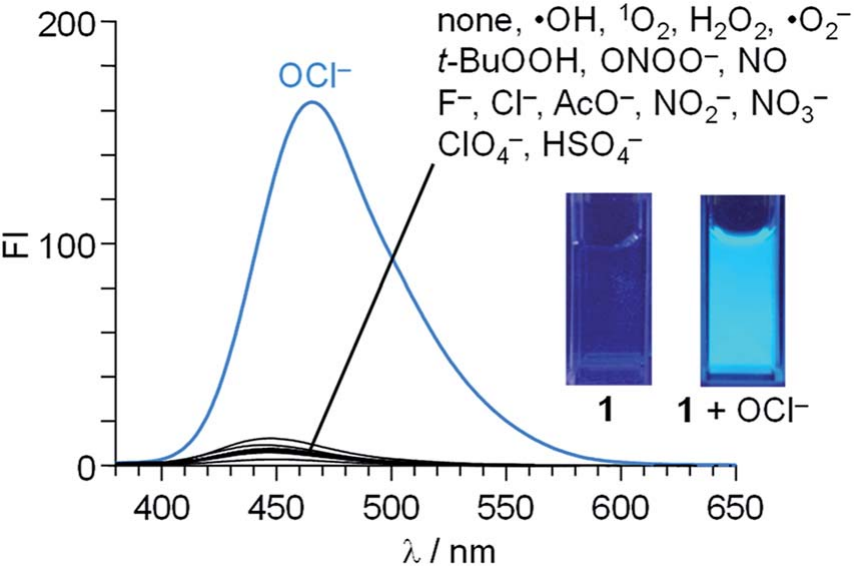
Fluorescence spectra (*λ*_ex_ = 344 nm) of 1 (10 μM) in a buffered water/MeCN mixture (99/1 v/v; HEPES 0.1 M, pH 7.0) at 25 °C with 50 equiv. of each respective analytes. All spectra were obtained after stirring the solution for 20 min.

Fluorescence spectra of 1 (10 μM) were measured in a buffered water/MeCN mixture (99/1 v/v) with pH 7.0 (HEPES 0.1 M) at 25 °C (*λ*_ex_ = 344 nm). As shown in Fig. 1, 1 itself shows a very weak fluorescence (fluorescence quantum yield, *Φ*_F_ = 0.002). In contrast, addition of 50 equiv. of OCl^−^ to the solution followed by stirring for 20 min creates a strong blue fluorescence at 462 nm (*Φ*_F_ = 0.082). Other anions (F^−^, Cl^−^, AcO^−^, NO_2_^−^, NO_3_^−^, ClO_4_^−^, and HSO_4_^−^), ROS [hydroxyl radical (·OH), singlet oxygen (^1^O_2_), H_2_O_2_, superoxide radical (·O_2_^−^), and *tert*-butyl hydroperoxide (*t*-BuOOH)], or RNS [NO and peroxynitrite (ONOO^−^)], when added to the solution containing 1, scarcely change the fluorescence spectra, indicating that OCl^−^ selectively triggers fluorescence enhancement of 1.

**Fig. 2:**
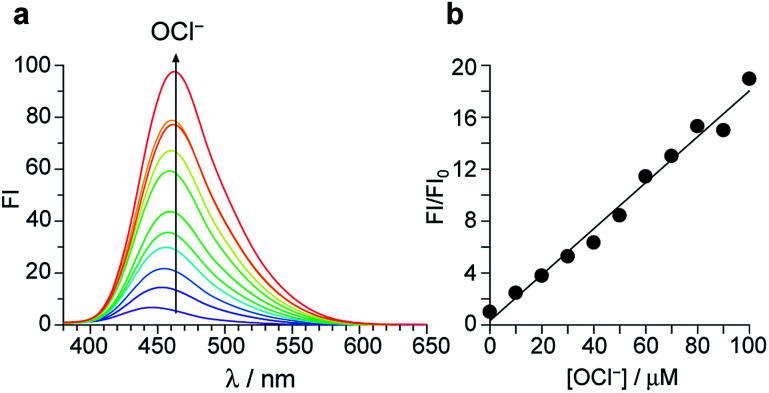
(a) Change in fluorescence spectra of 1 (10 μM) upon titration with OCl^−^ in a buffered water/MeCN mixture (99/1 v/v; HEPES 0.1 M, pH 7.0) at 25 °C. (b) Change in the ratio of fluorescence intensity at 462 nm (FI/FI_0_) *versus* the OCl^−^ concentration. The respective data were obtained after stirring the solution for 20 min.

Fig. 2a shows the results of fluorescence titration of 1 with OCl^−^. Stepwise addition of OCl^−^ increases the intensity of the 462 nm fluorescence. As shown in Fig. 2b, the change in the ratio of fluorescence intensity at 462 nm (FI/FI_0_) with the OCl^−^ concentrations clearly shows linear relationship, indicating that 1 facilitates accurate OCl^−^ sensing at ∼100 μM. The lower detection limit was determined to be 3.3 μM based on the signal-to-noise (S/N) ratio using the equation (DL = 3 × SD/*S*),^26^ where SD is the standard deviation of blank analysis (SD = 0.19, *n* = 10) and *S* is the slope of the fluorescence intensity *versus* the OCl^−^ concentrations (*S* = 0.18 μM^−1^). This detection limit (3.3 μM) is lower than the physiological OCl^−^ concentrations (5–25 μM) in the human body,^27^ suggesting that 1 facilitates sensitive OCl^−^ detection even in high-water-content solution.

### Reaction of the sensor with OCl^−^

**Fig. 3:**
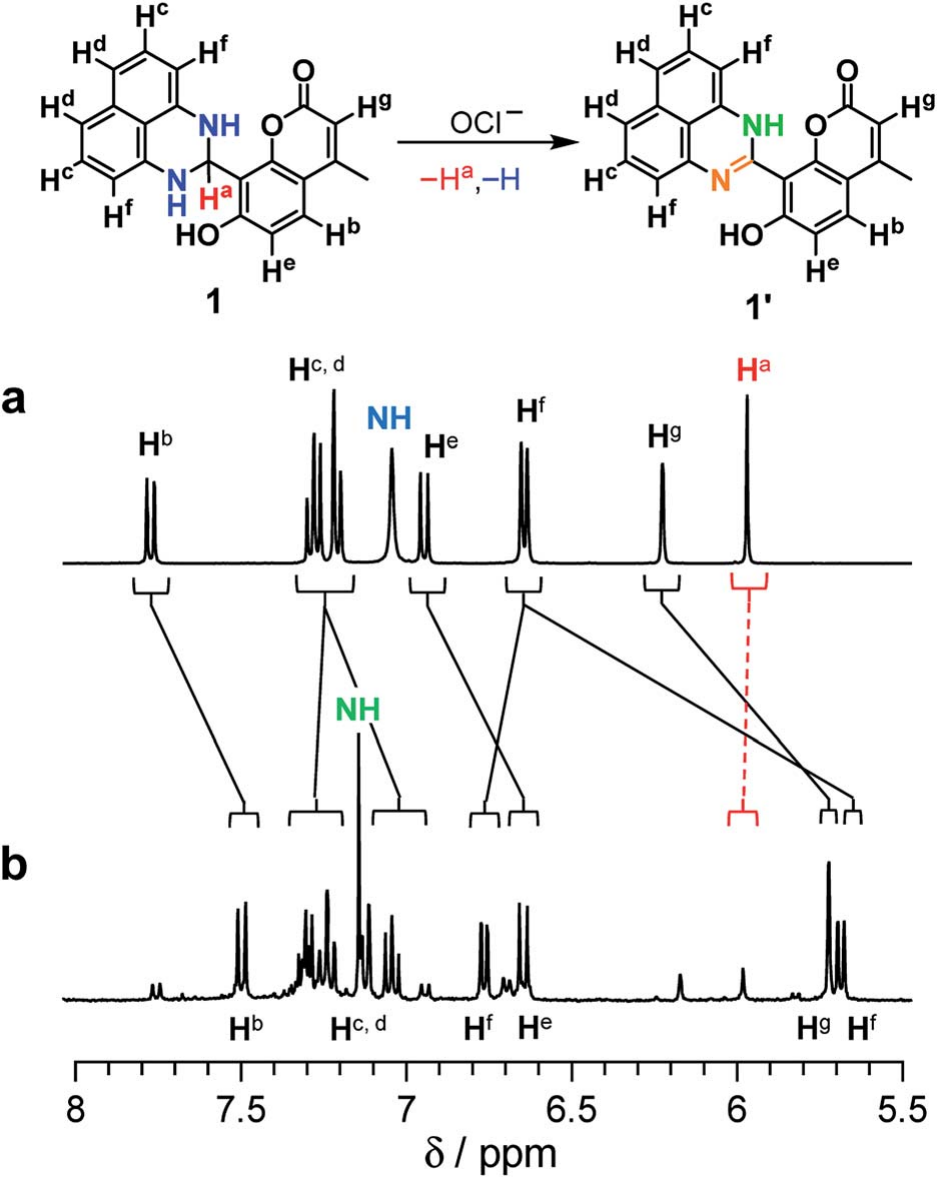
^1^H NMR chart of 1 (24 mM) measured in DMSO-d_6_ (a) without and (b) with 8 equiv of OCl^−^ (400 MHz, 30 °C).

As shown in Scheme 2, the turn-on fluorescence response of 1 upon addition of OCl^−^ is triggered by the transformation to 1′, *via* dehydrogenation of the dihydroperimidine moiety of 1. This transformation is confirmed by ^1^H, ^13^C NMR and FAB-MS analysis of a DMSO-d_6_ solution containing 1 and OCl^−^ (Fig. S5–S7, ESI†). Partial ^1^H NMR charts of 1 and 1′ measured in DMSO-d_6_ are shown in Fig. 3, where the 2D COSY spectra were used for the assignment of the respective chemical shifts (Fig. S8 and S9, ESI†). As shown in Fig. 3a, 1 shows an H^a^ proton at the 2-position of the dihydroperimidine unit at 6.0 ppm. However, as shown in Fig. 3b, addition of OCl^−^ to the solution leads to almost complete disappearance of the H^a^ proton. In addition, 1 shows two N–H protons of the dihydroperimidine moiety at 7.0 ppm. After the addition of OCl^−^, its chemical shift moves to 7.1 ppm, and its integral value becomes almost 1. These data indicate that H^a^ and one N–H proton of 1 are removed by the reaction with OCl^−^. The dehydrogenation of 1 by OCl^−^ is confirmed by FAB-MS analysis. As shown in Fig. S3 (ESI†), 1 shows a peak at *m*/*z* 344.1 assigned to [1]^+^. In contrast, as shown in Fig. S7 (ESI†), a solution containing 1 and OCl^−^ shows a peak at *m*/*z* 342.1 assigned to the dehydrogenated product [1′]^+^. These NMR and FAB-MS data clearly suggest that dehydrogenation of the dihydroperimidine moiety of 1*via* the oxidation by OCl^−^ gives 1′ containing the perimidine moiety.

**Fig. 4:**
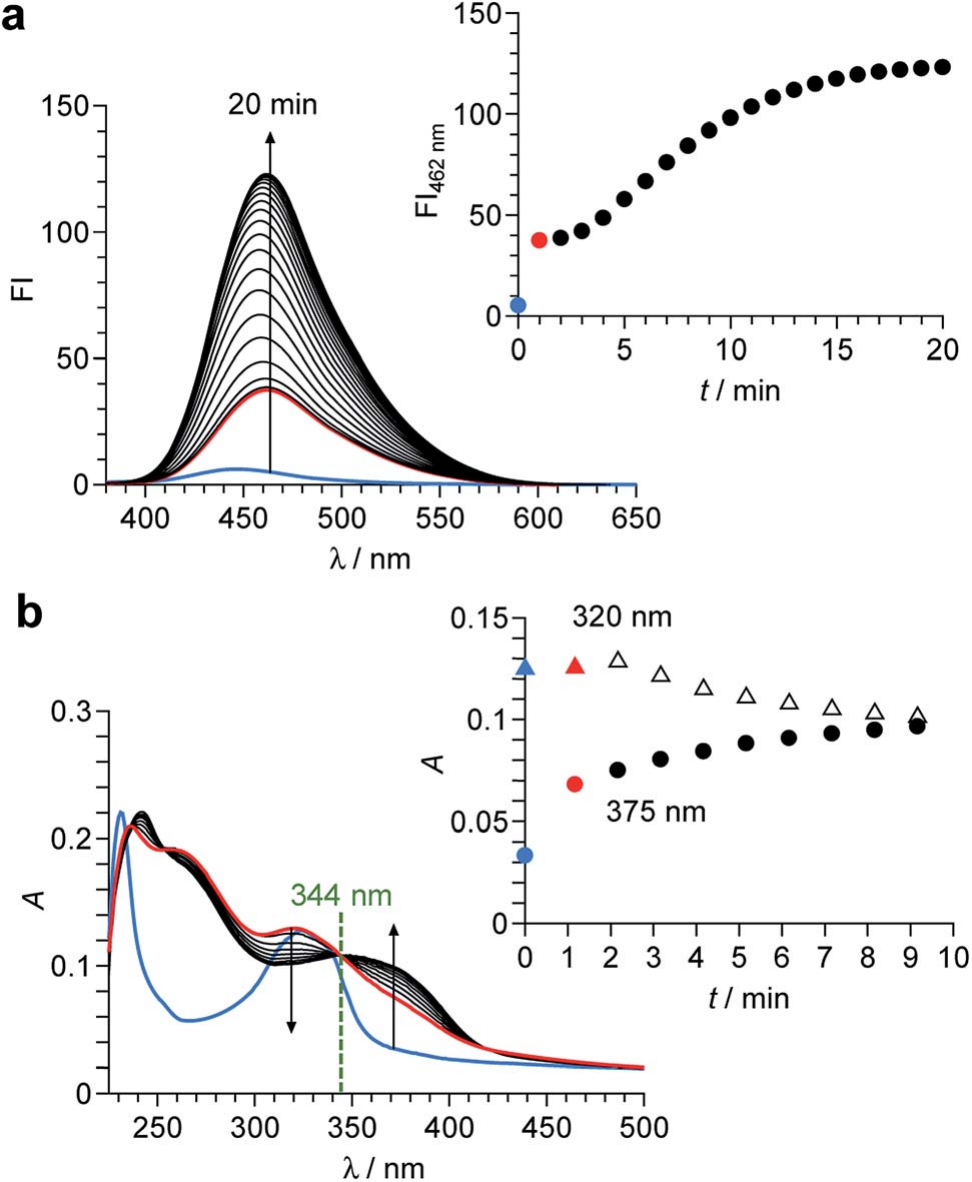
(a) Time-dependent change in fluorescence spectra (*λ*_ex_ = 344 nm) of 1 (10 μM) in a buffered water/MeCN mixture (99/1 v/v; HEPES 0.1 M, pH 7.0) at 25 °C after addition of 50 equiv. of OCl^−^. The inset shows change in the intensity at 462 nm. (b) Time-dependent change in absorption spectra of 1 after addition of 50 equiv. of OCl^−^. The inset shows change in the absorbance at 320 nm and 375 nm.

As shown in Scheme 2, the enol–imine form (1′) is rapidly produced by dehydrogenation of 1 by OCl^−^ and shows a weak fluorescence (*Φ*_F_ = 0.009). Then, 1′ undergoes tautomerization to the keto-amine form (1′′) *via* a proton transfer of the coumarin –OH to the imine nitrogen of the perimidine unit, as often observed for similar *o*-hydroxyl Schiff bases,^28,29^ and exhibits a strong fluorescence (*Φ*_F_ = 0.082). This sequence is confirmed by time-dependent changes in the absorption and fluorescence spectra of 1 monitored after addition of OCl^−^. As shown in Fig. 4a, addition of OCl^−^ immediately increases the fluorescence intensity at 462 nm within 1 min (blue to red line), although the intensity is weak. Then, the intensity gradually increases with time and plateaus after 15 min, creating a strong fluorescence, where the emission wavelengths scarcely change during the measurements. This indicates that the reaction of 1 with OCl^−^ creates two different emitting species. As shown in Fig. 4b, absorption spectrum of 1 also changes immediately after the OCl^−^ addition within 1 min (blue to red line). Then, the spectrum changes gradually with a decrease in *ca.* 320 nm absorbance and an increase in *ca.* 375 nm absorbance. The isosbestic point at 344 nm clearly indicates that, as shown in Scheme 2, the reaction of 1 with OCl^−^ rapidly produces a weakly-fluorescent enol-imine form (1′) and its slow tautomerization by the intramolecular proton transfer creates a strongly-fluorescent keto-amine form (1′′).

The tautomerization of 1′ to 1′′ is promoted by polar water molecules. It is well known that, for the tautomerization of *o*-hydroxy Schiff bases,^30,31^ the enol-imine form is stabilized in less polar solvents such as benzene, while the keto-amine form is stable in polar solvents such as EtOH. Fig. S10 (ESI†) shows the change in fluorescence intensity of 1 after addition of OCl^−^ in MeCN solutions with different water contents. In all solutions, the weakly-fluorescent enol-imine form (1′) is rapidly produced by the addition of OCl^−^. The strongly-fluorescent keto-amine form (1′′) is not produced in low-water-content solutions (30% and 60%), whereas increasing the water content produces 1′′. This indicates that increasing water content increases the polarity of solutions and promotes 1′-to-1′′ tautomerization. However, as shown in Fig. 3b and S5–S7 (ESI†), ^1^H, ^13^C NMR and FAB-MS analysis of the product obtained by the reaction of 1 with OCl^−^ in DMSO-d_6_ detected 1′. This is because the 1′-to-1′′ tautomerization is not promoted in less polar DMSO. These findings clearly support the 1 → 1′ → 1′′ transformation by the reaction of 1 with OCl^−^ in high-water-content solutions, as shown in Scheme 2.

### *Ab initio* calculations

**Fig. 5:**
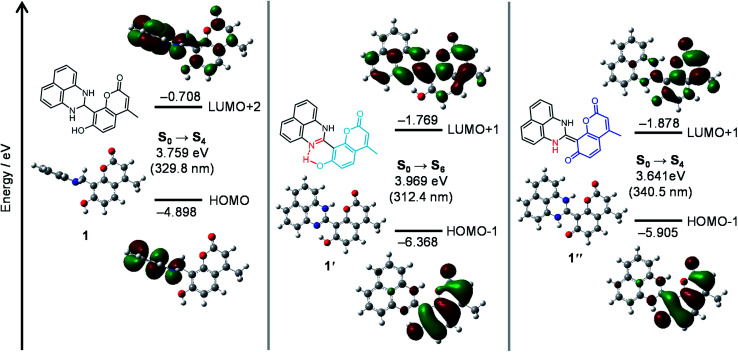
Energy diagrams and interfacial plots of main molecular orbitals of (left) 1, (center) 1′ and (right) 1′′, calculated at the DFT level (B3LYP/6-31+G*).

The mechanism for the turn-on fluorescence response of 1 was clarified by *ab initio* calculations. The structures and optical properties of 1, 1′, and 1′′ species were calculated by the density functional theory (DFT) and the time-dependent DFT (TD-DFT), respectively, within the Gaussian 03 program with water as a solvent. As summarized in Table S1 (ESI†), singlet electronic transition of 1 mainly consists of HOMO → LUMO+2 (S_0_ → S_4_) transition. Its calculated transition energy (3.76 eV, 330 nm) is close to the absorption maximum (*λ*_max_) of 1 at 323 nm (Fig. 4b, blue line). As shown in Fig. 5 (left), π-electrons of both HOMO and LUMO+2 of 1 are localized on the dihydroperimidine moiety, indicating that photoexcitation of the coumarin fluorophore is not populated. This therefore results in almost no fluorescence of 1.

As shown in Fig. 5 (center), optimized structure of 1′ has a planar structure, where the coumarin and perimidine units lie on the same plane. It is noted that the structural optimization spontaneously creates an H-bonding interaction between the imine nitrogen and coumarin –OH units, in which the N–O distance of ∼2.5 Å indicates strong electrostatic interaction between these units.^32^ The structural regulation by the H-bonding may create the planer structure. As shown in Table S1 (ESI†), the electronic transition of 1′ mainly consists of HOMO-1 → LUMO+1 (S_0_ → S_6_) transition. Its energy (3.97 eV, 312 nm) is also close to that for the absorption maximum (320 nm) of 1′ (Fig. 4b, red line). As shown in Fig. 5 (center), relatively large distribution of π-electrons are observed on both HOMO-1 and LUMO+1 for 1′. This is because the H-bonding interaction of the coumarin –OH increases the electron density of coumarin unit.^33^ The enhanced photoexcitation of the coumarin units may therefore result in weak fluorescence of 1′.

As shown in Fig. 5 (right), optimized structure of 1′′ also has a planar structure owing to the C=C bond formation between the coumarin and dihydroperimidine units. Singlet electronic transition of 1′′ is mainly contributed by HOMO-1 → LUMO+1 (S_0_ → S_4_) transition (Table S1, ESI†). Its transition energy (3.64 eV, 340 nm) is also close to that for the absorption band (375 nm) of 1′′ (Fig. 4b). As shown in Fig. 5 (right), almost all of the π-electrons of both HOMO-1 and LUMO+1 for 1′′ are localized on the coumarin units because complete deprotonation of the coumarin –OH significantly increases the electron density of the coumarin unit.^33^ This therefore results in strong coumarin fluorescence from 1′′.

The total energies of 1′ and 1′′ in water were determined to be −717846.94 and −717850.01 kcal mol^−1^, respectively. The lower energy of 1′′ (Δ*E* = 3.07 kcal mol^−1^) indicates that the keto-amine form (1′′) is indeed more stable in water than the enol-imine form (1′). This further supports the 1 → 1′ → 1′′ transformation by the reaction of 1 with OCl^−^ in high-water-content solutions. These DFT results clearly indicate that the dihydroperimidine unit acts as a proton acceptor for the coumarin –OH (Scheme 2). The OCl^−^-triggered formation of the perimidine unit leads to H-bonding interaction between the imine nitrogen and coumarin –OH and creates weak emission (1′). Water-assisted tautomerization of 1′ to 1′′ leads to complete proton transfer from the coumarin –OH and creates strong emission (1′′).

### Effect of pH

**Fig. 6:**
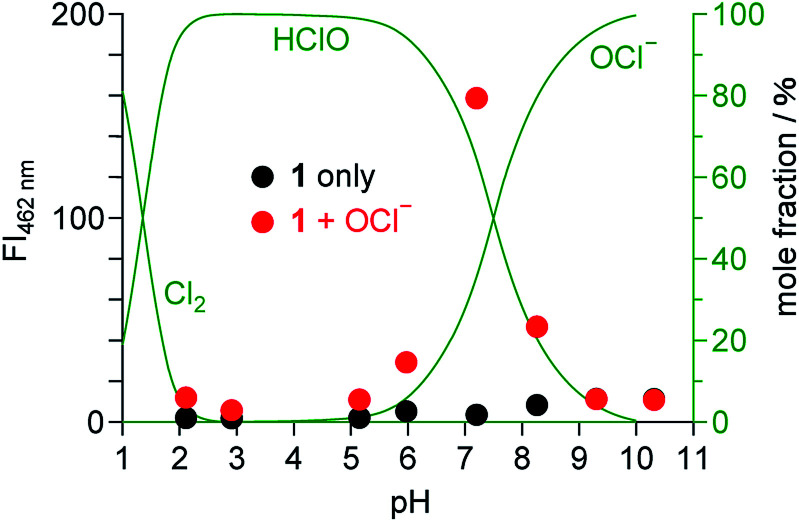
Fluorescence intensity of 1 (10 μM) monitored at 462 nm in water/MeCN mixtures (99/1 v/v) at 25 °C with different pH, (red) with and (black) without OCl^−^ (50 equiv.). The mole fraction distributions of Cl_2_, HClO, and OCl^−^ calculated based on the equilibria (eqn (1) and (2)) are shown by green lines.

It is noted that pH of the solution is critical for the OCl^−^ sensing. Fig. 6 shows the fluorescence intensity of 1 at 462 nm measured at different pH with and without 50 equiv. of OCl^−^, where the mole fraction distributions of Cl_2_, HClO, and OCl^−^ are also shown based on their equilibria in water,^34,35^ using the following equations:

1 Cl_2_ + H_2_O ⇌ HClO + H^+^ + Cl^−^ (p*K*_a_ = 1.4)

2 HClO ⇌ ClO^−^ + H^+^ (p*K*_a_ = 7.6)

The fluorescence enhancement of 1 by OCl^−^ occurs at neutral physiological pH (6–8), and does not occur at acidic or basic pH. In acidic media (pH < 6), protonation of OCl^−^ (HClO formation; eqn (2)) cancels the basicity of OCl^−^ and, hence, suppresses dehydrogenation of the dihydroperimidine unit of 1. In contrast, basic media (pH > 8) stabilize OCl^−^, but the fluorescence enhancement does not occur. This is probably because, as observed for several OCl^−^ sensors,^10,14,15^ the oxidation ability of OCl^−^ decreases in basic media and inhibits dehydrogenation of the dihydroperimidine unit. These data suggest that 1 facilitates fluorometric sensing of OCl^−^ in physiological pH media (pH 6–8).

## Conclusions

We synthesized a coumarin–dihydroperimidine dye (1), acting as a fluorescent sensor for OCl^−^ in 99% water. 1 shows a weak fluorescence, but OCl^−^-selective dehydrogenation of its dihydroperimidine unit creates a strong blue fluorescence. 1 facilitates selective and sensitive OCl^−^ detection at physiological pH. The turn-on response of 1 occurs *via* two-step reactions. The dehydrogenation by OCl^−^ rapidly produces the enol-imine form (1′) involving the H-bonding interaction between the imine nitrogen and coumarin –OH. This increases the electron density of the coumarin unit, resulting in weak fluorescence. 1′ undergoes tautomerization to the keto-amine form (1′′) due to the stabilization in polar water media. The complete proton transfer from the coumarin –OH to the imine nitrogen significantly increases the electron density of the coumarin unit, exhibiting a strong fluorescence. The molecular design based on the dihydroperimidine unit as an OCl^−^-driven proton sensor, may contribute to the design of efficient fluorescent sensors for OCl^−^ in environmental and biological samples.

## Experimental

### General

All chemicals were used as received. ·OH was generated by the Fenton reaction.^36^^1^O_2_ was generated from the H_2_O_2_/MoO_4_^2−^ system in alkaline media.^37^ NO was generated using sodium nitroferricyanide(iii) dehydrate.^38^ ONOO^−^ was generated from the SIN-1 reagent (Dojindo Molecular Technologies, Japan). ·O_2_^−^ was generated using potassium superoxide (KO_2_).^36^ Fluorescence spectra were measured on a JASCO FP-6500 fluorescence spectrophotometer with a 10 nm path length cell (both excitation and emission slit widths, 5.0 nm) at 298 ± 1 K using a temperature controller.^39^ Absorption spectra were measured on an UV-visible photodiode-array spectrometer (Shimadzu; Multispec-1500) equipped with a temperature controller (S-1700).^40^ All measurements were performed under aerated conditions. ^1^H and ^13^C NMR charts were obtained using a JEOL JNM-ECS400 spectrometer. FAB-MS analysis was performed on a JEOL JMS 700 Mass Spectrometer. Fluorescence quantum yields (*Φ*_F_) were determined with quinine sulfate dihydrate (in 0.1 M HClO_4_ solution) as a standard.^41,42^

### Synthesis of the sensor (1) [8-(2,3-dihydro-1*H*-perimidin-2-yl)-7-hydroxy-4-methyl-2*H*-chromen-2-one]

8-Formyl-7-hydroxy-4-methylcoumarin (200 mg, 0.98 mmol)^25^ and 1,8-diaminonaphthalene (188 mg, 1.20 mmol) were dissolved in EtOH (20 ml), and the solution was stirred at 80 °C for 2.5 h. The solid formed was recovered by filtration and washed thoroughly with EtOH, affording 1 as pale pink solids. Yield: 234.4 mg (69.4%). ^1^H NMR (400 MHz, DMSO-d_6_, TMS), *δ* (ppm): 10.38 (1H, s), 7.74 (1H, d, *J* = 8.8 Hz), 7.24–7.28 (2H, m), 7.18–7.20 (2H, m), 7.03 (2H, s), 6.94 (1H, d, *J* = 8.8 Hz), 6.64 (2H, d, *J* = 7.2 Hz), 6.23 (1H, s), 5.99 (1H, s), 2.44 (3H, s). ^13^C NMR (100 MHz, DMSO-d_6_, TMS), *δ* (ppm): 161.0, 159.6, 153.8, 152.4, 142.9, 134.2, 126.9, 126.7, 117.2, 113.5, 113.2, 112.0, 110.9, 110.3, 106.3, 59.9, 55.9, 18.3. FAB-MS: *m*/*z*: calcd for C_21_H_16_O_3_N_2_^+^ (M^+^) 344.1161; found (ESI†): 344.1158.

### Calculation details

*Ab initio* calculations were performed with tight convergence criteria at the DFT level within the Gaussian 03 package, using the B3LYP/6-31+G(D) basis set for all atoms. The excitation energies and oscillator strengths of the compounds were calculated by TDTFT^43^ at the same level of optimization using the PCM with water as a solvent.^44^ Cartesian coordinates are summarized at the end of ESI.†

## Conflicts of interest

There are no conflicts to declare.

## Supplementary Material

RA-009-C9RA05533A-s001
