# Supplementary material for: A coumarin–dihydroperimidine dye as a fluorescent chemosensor for hypochlorite in 99% water
Source: RSC Adv. 2019 Sep 11;9(49):28636–41. doi: 10.1039/c9ra05533a (PMC9071195; doi:10.1039/c9ra05533a)
Supplement: RA-009-C9RA05533A-s001 [file RA-009-C9RA05533A-s001.pdf]

Electronic Supplementary Material (ESI) for RSC Advances.
This journal is © The Royal Society of Chemistry 2019

Electronic Supplementary Information (ESI†)

A coumarin–dihydroperimidine dye as a fluorescent chemosensor for hypochlorite in 99% water

Yasuhiro Shiraishi,* Chiharu Yamada and Takayuki Hirai

Research Center for Solar Energy Chemistry, and Division of Chemical Engineering, Graduate School of Engineering Science, Osaka University, Toyonaka 560-8531, Japan

shiraish@cheng.es.osaka-u.ac.jp

CONTENTS

Page
Table S1 TD-DFT calculation results for **1, 1′** and **1″** 2
Fig. S1 ^{1}H NMR chart of **1** 4
Fig. S2 ^{13}C NMR chart of **1** 5
Fig. S3 FAB-MS chart of **1** 6
Fig. S4 Absorption spectra of **1** 7
Fig. S5 ^{1}H NMR chart of **1′** 8
Fig. S6 ^{13}C NMR chart of **1′** 9
Fig. S7 FAB-MS chart of **1′** 10
Fig. S8 ^{1}H–^{1}H COSY chart of **1** 11
Fig. S9 ^{1}H–^{1}H COSY chart of **1′** 12
Fig. S10 Effect of water content on the fluorescence intensity of **1** 13
Cartesian coordinates for respective compounds 14

Table S1. Calculated excitation energy (*E*), wavelength (λ), and oscillator strength (*f*) for low-laying singlet state (S_n) of **1**, **1′** and **1′′**.[a]

compound		Main orbital transition (CIC [b])	*E* (eV) [λ (nm)]	*f*
**1**	$S_0 \rightarrow S_1$	HOMO → LUMO (0.70571)	2.2918 eV [540.99 nm]	0.0023
	$S_0 \rightarrow S_2$	HOMO-1 → LUMO (0.70440)	3.0942 eV [400.70 nm]	0.0000
	$S_0 \rightarrow S_3$	HOMO → LUMO+1 (0.70406)	3.3076 eV [374.84 nm]	0.0008
	$S_0 \rightarrow S_4$	HOMO → LUMO+2 (0.65062)<br>HOMO → LUMO+4 (-0.10429)	3.7590 eV [329.83 nm]	0.1681
	$S_0 \rightarrow S_5$	HOMO → LUMO+3 (0.66998)<br>HOMO → LUMO+5 (-0.13878)<br>HOMO → LUMO+6 (-0.12101)	3.8658 eV [320.72 nm]	0.0014
	$S_0 \rightarrow S_6$	HOMO-2 → LUMO (0.67869)<br>HOMO → LUMO+5 (-0.16381)	3.9242 eV [315.95 nm]	0.0031
**1′**	$S_0 \rightarrow S_1$	HOMO → LUMO (0.67202)<br>HOMO → LUMO+1 (0.15556)	2.5622 eV [483.90 nm]	0.0271
	$S_0 \rightarrow S_2$	HOMO → LUMO (-0.18684)<br>HOMO → LUMO+1 (0.66377)	2.8956 eV [428.17 nm]	0.0152
	$S_0 \rightarrow S_3$	HOMO-2 → LUMO+3 (-0.10524)<br>HOMO-1 → LUMO+1 (0.12636)<br>HOMO → LUMO+2 (0.62423)<br>HOMO → LUMO+3 (-0.10333)	3.7678 eV [329.06 nm]	0.2785
	$S_0 \rightarrow S_4$	HOMO-3 → LUMO (0.13294)<br>HOMO-3 → LUMO+4 (-0.11075)<br>HOMO-1 → LUMO (0.58062)<br>HOMO-1 → LUMO+1 (0.26085)	3.8169 eV [324.83 nm]	0.172
	$S_0 \rightarrow S_5$	HOMO-2 → LUMO+2 (0.20599)<br>HOMO-1 → LUMO+1 (0.29346)<br>HOMO → LUMO+3 (0.56823)	3.9169 eV [316.53 nm]	0.0572
	$S_0 \rightarrow S_6$	HOMO-3 → LUMO (0.14907)<br>HOMO-2 → LUMO+2 (-0.11040)<br>HOMO-1 → LUMO (-0.26941)<br>HOMO-1 → LUMO+1 (0.51253)<br>HOMO → LUMO+2 (-0.13810)<br>HOMO → LUMO+3 (-0.24763)	3.9693 eV [312.36 nm]	0.2865
**1′′**	$S_0 \rightarrow S_1$	HOMO → LUMO (0.66986)	2.8187 eV [439.87 nm]	0.0095

	HOMO → LUMO+1 (0.12792)		
$S_0 \rightarrow S_2$	HOMO → LUMO (-0.14003)<br>HOMO → LUMO+1 (0.68548)	3.2523 eV [381.22 nm]	0.0010
$S_0 \rightarrow S_3$	HOMO-1 → LUMO (0.59565)<br>HOMO-1 → LUMO+1 (0.29465)	3.2670 eV [379.50 nm]	0.2737
$S_0 \rightarrow S_4$	HOMO-1 → LUMO (-0.27485)<br>HOMO-1 → LUMO+1 (0.58007)	3.6413 eV [340.50 nm]	0.3661
$S_0 \rightarrow S_5$	HOMO-7 → LUMO (0.10009)<br>HOMO-3 → LUMO+3 (0.11442)<br>HOMO → LUMO+2 (0.63331)	3.8806 eV [319.49 nm]	0.1753
$S_0 \rightarrow S_6$	HOMO-3 → LUMO+1 (-0.29114)<br>HOMO-1 → LUMO+2 (-0.11142)<br>HOMO → LUMO+3 (0.61780)	4.0682 eV [304.76 nm]	0.1186

[a] The optimized structures for the respective models are summarized in the end of this ESI.

[b] CI expansion coefficients for the main transitions.

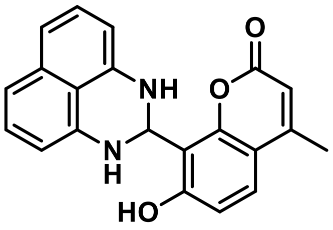

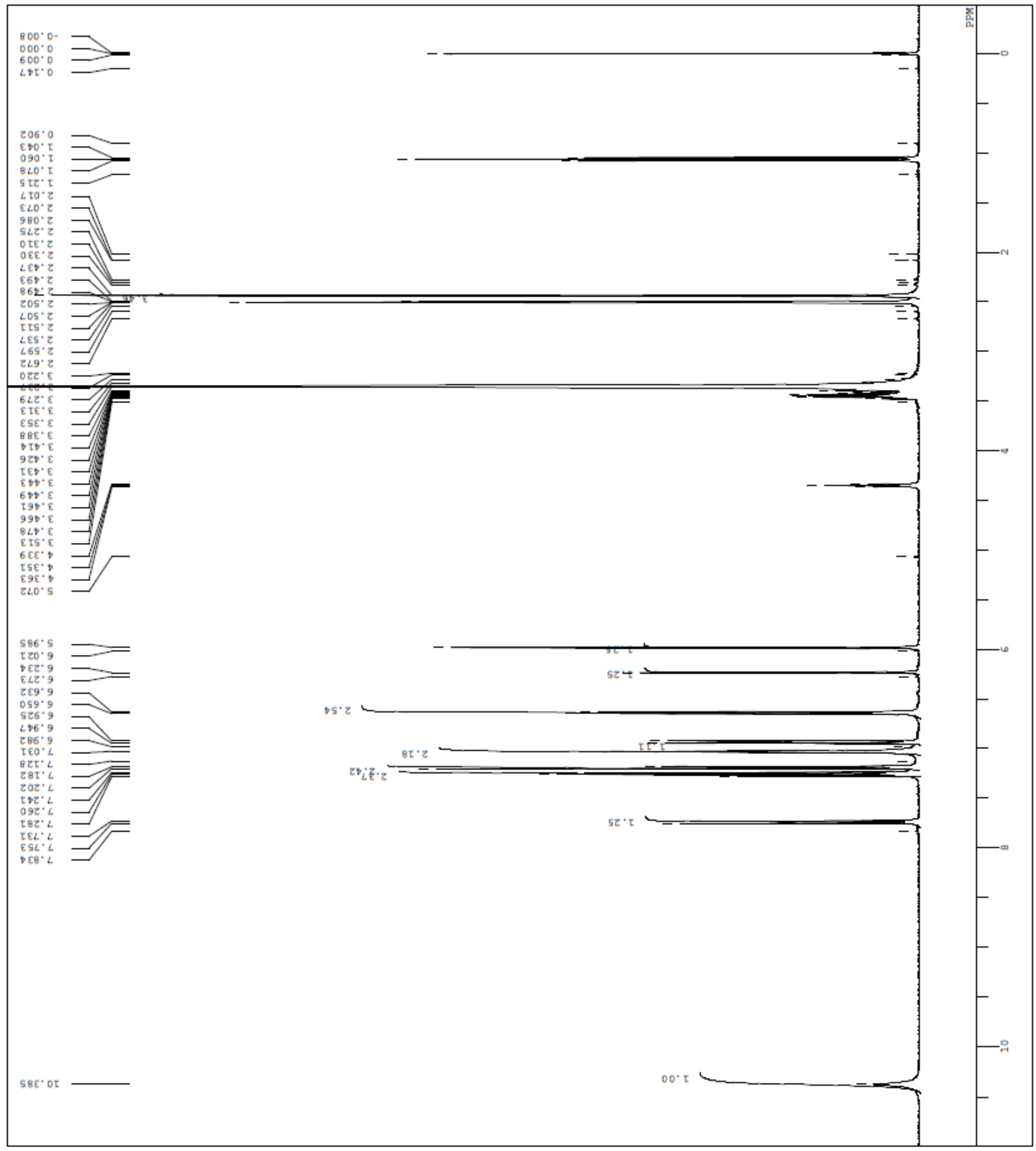

Fig. S1 ^{1}H NMR chart of **1** (24 mM, DMSO–d_6, 400 MHz).

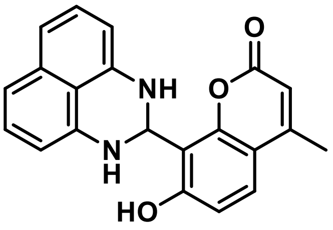

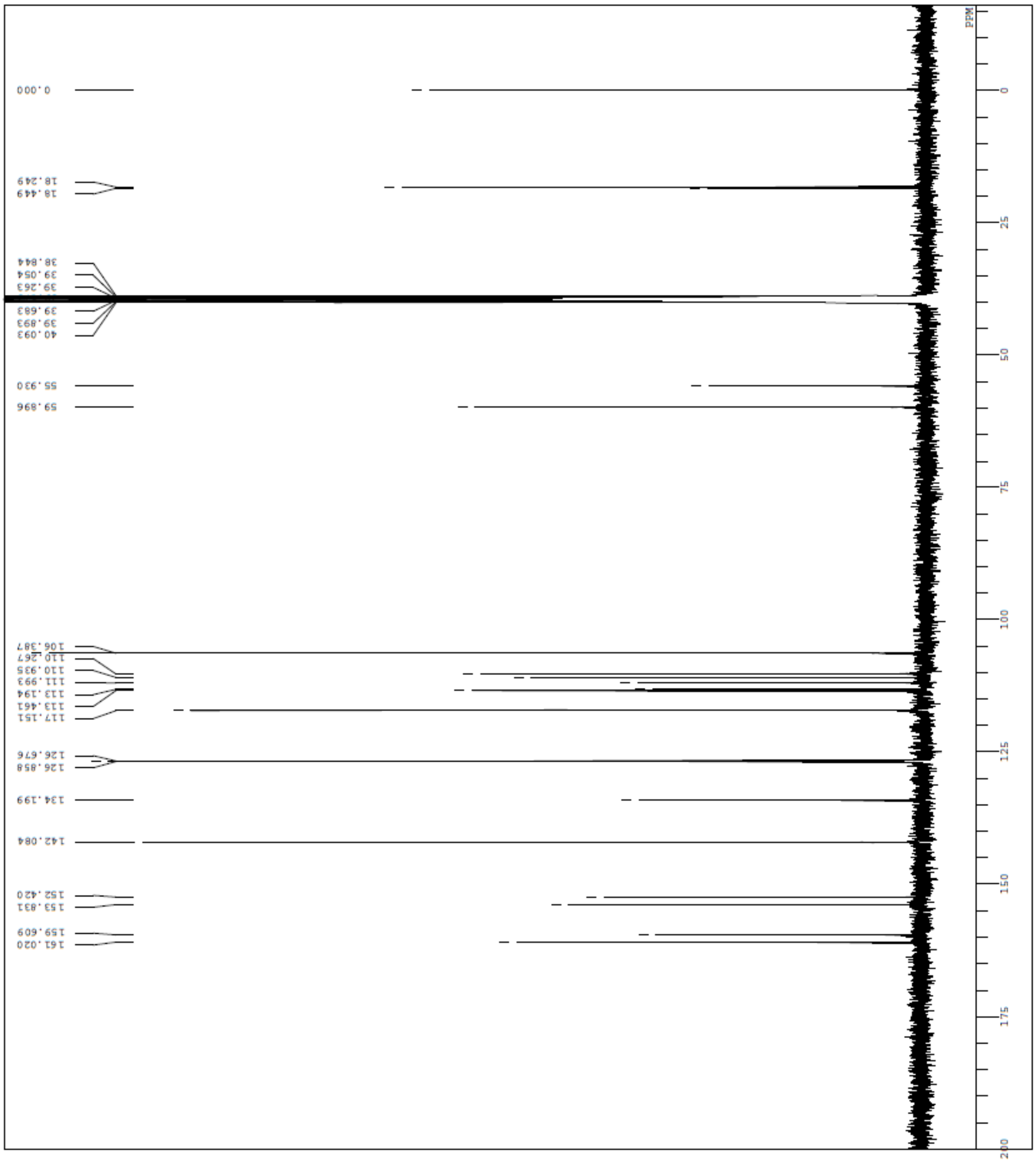

Fig. S2 ^{13}C NMR chart of **1** (48 mM, DMSO–d_6, 100 MHz).

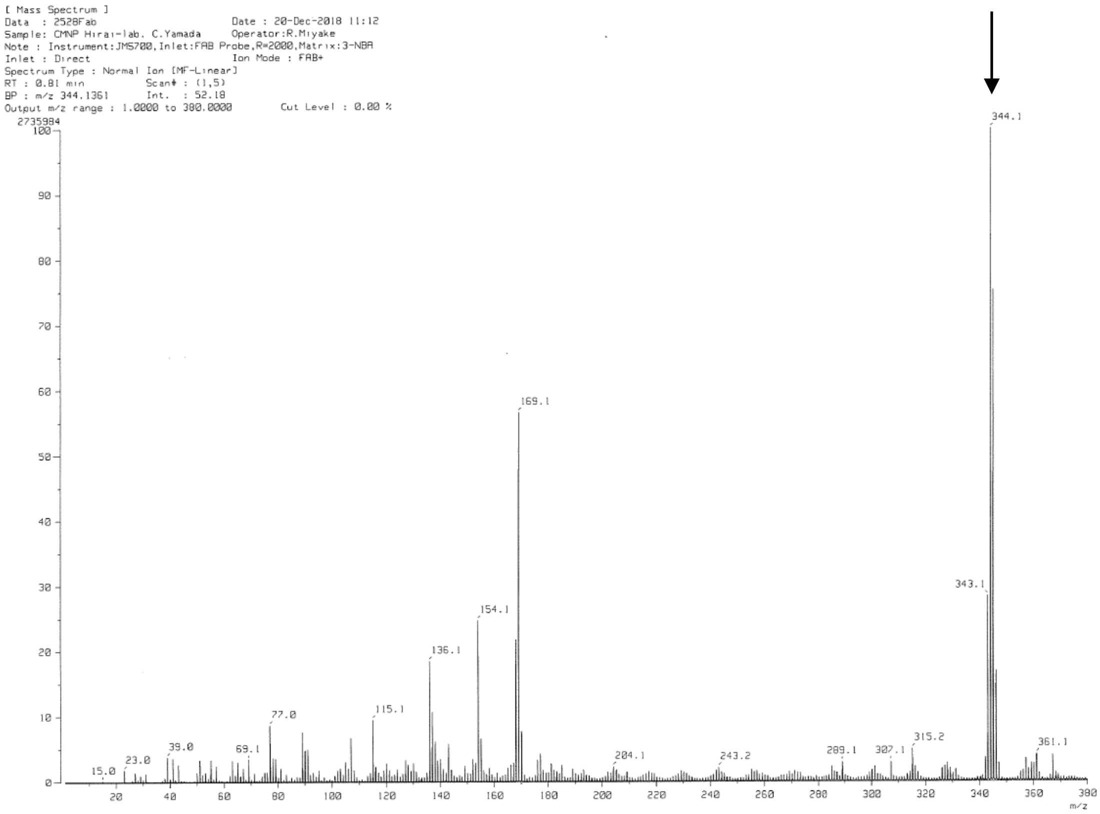

$C_{21}H_{16}O_3N_2^+$ (M^+) *m/z* 344.1158
(Calculated: $C_{21}H_{16}O_3N_2^+$ (M^+) *m/z* 344.1161)

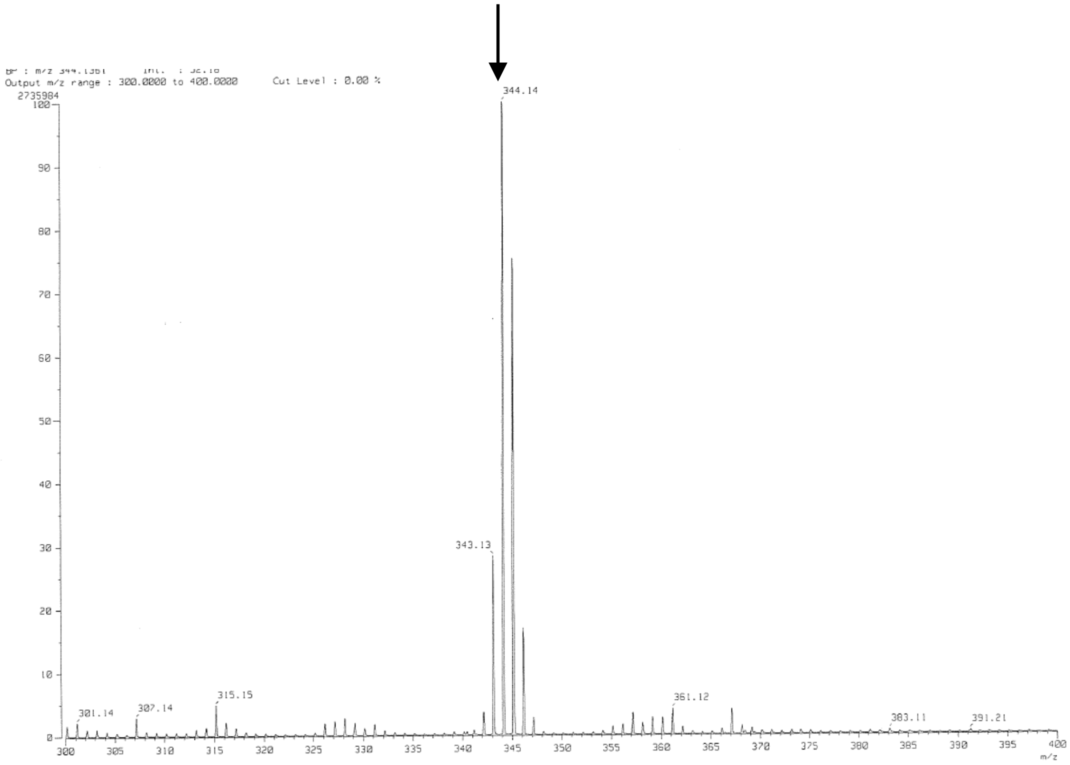

Fig. S3 FAB–MS chart of **1**. (top) full and (bottom) partial charts.

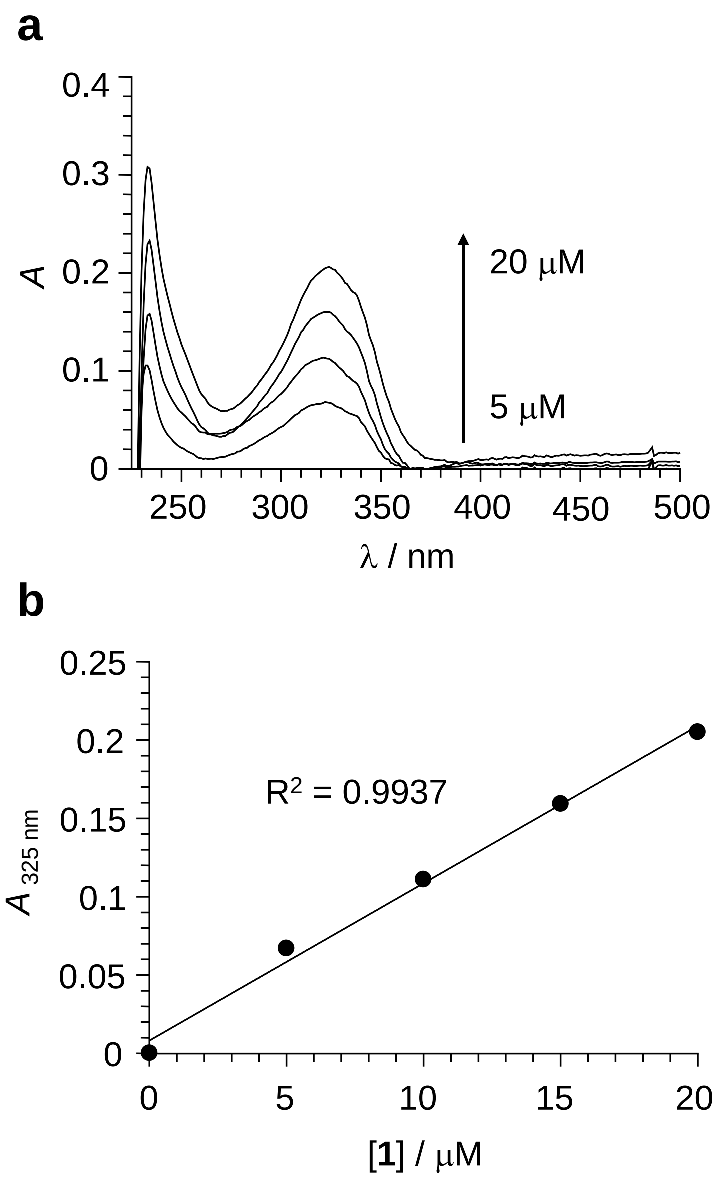

Fig. S4 (a) Change in absorption spectra of **1** measured at 25 °C in 1% MeCN solutions (HEPES 0.1 M, pH 7.0) with different concentration of **1**. (b) Change in the absorbance at 325 nm as a function of the concentration of **1**.

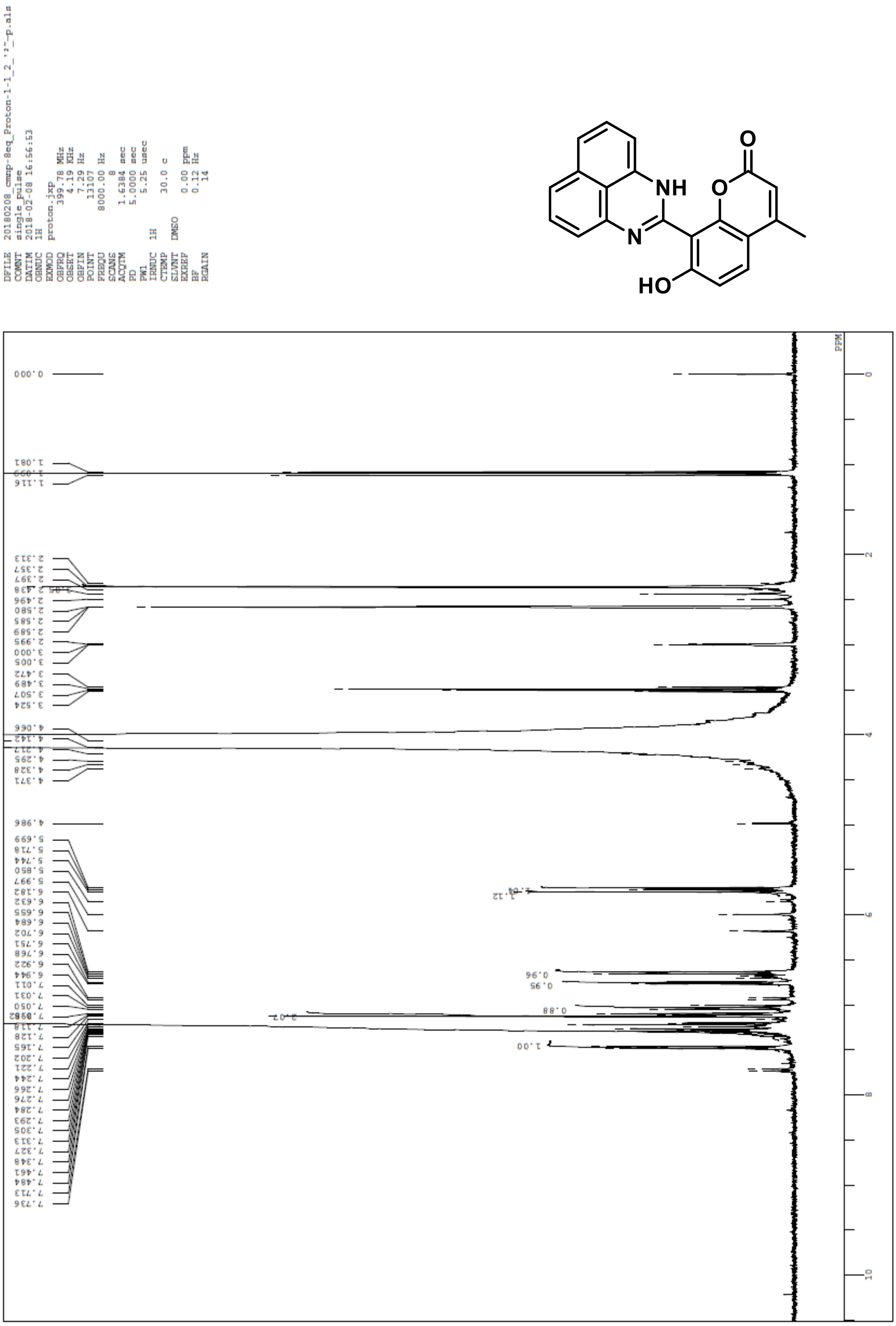

Fig. S5 ^{1}H NMR chart of **1′** (24 mM, DMSO–d_6, 400 MHz).

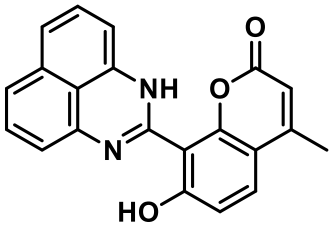

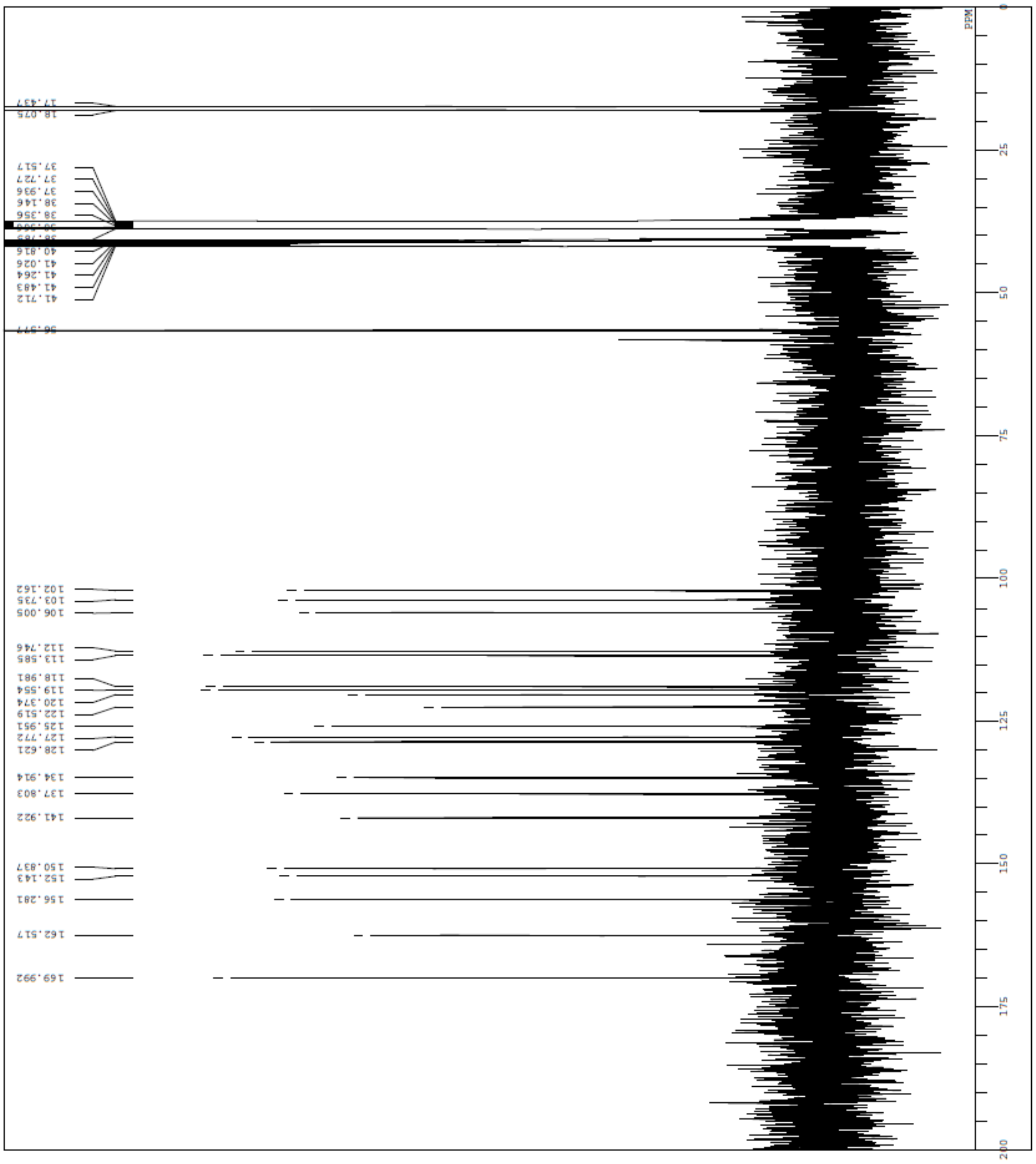

Fig. S6 ^{13}C NMR chart of **1′** (49 mM, DMSO–d_6, 100 MHz).

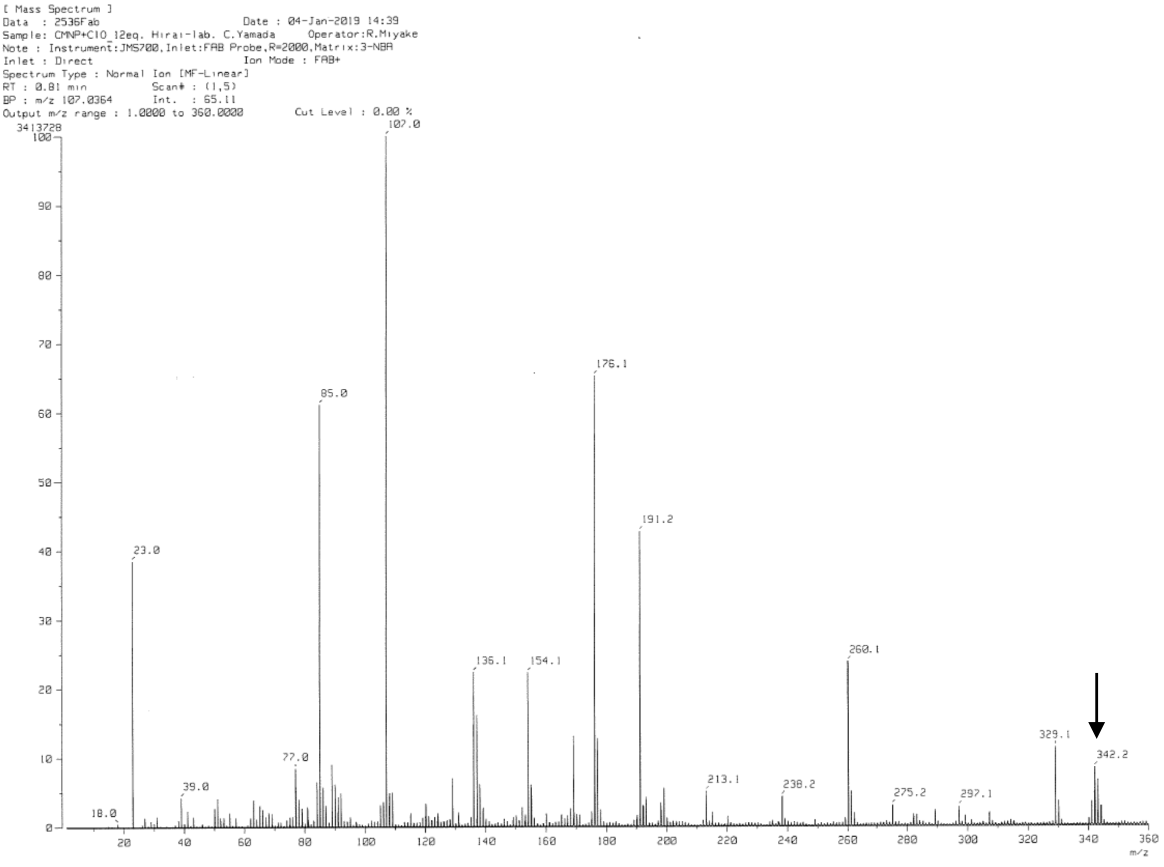

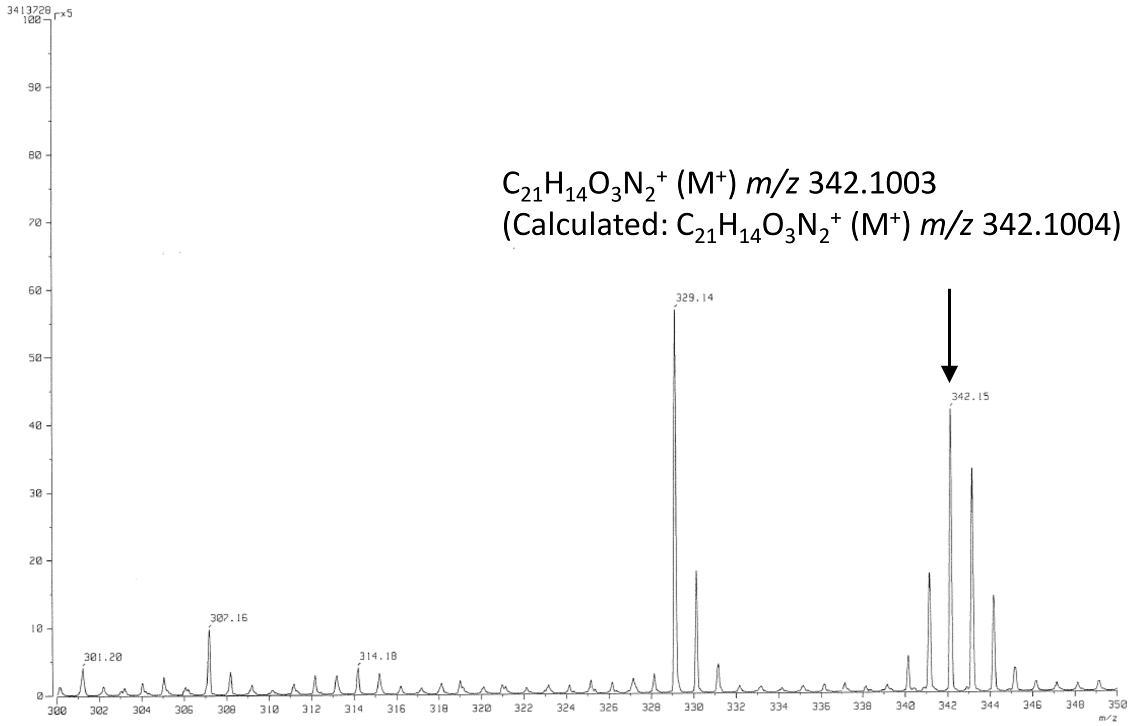

$C_{21}H_{14}O_3N_2^+$ (M^+) *m/z* 342.1003
(Calculated: $C_{21}H_{14}O_3N_2^+$ (M^+) *m/z* 342.1004)

Fig. S7 FAB–MS chart of **1′**. (top) full and (bottom) partial charts.

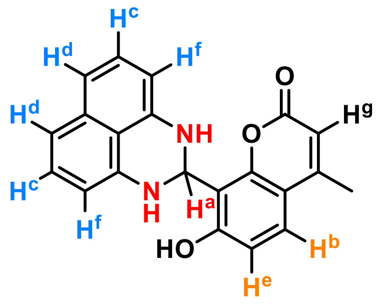

1

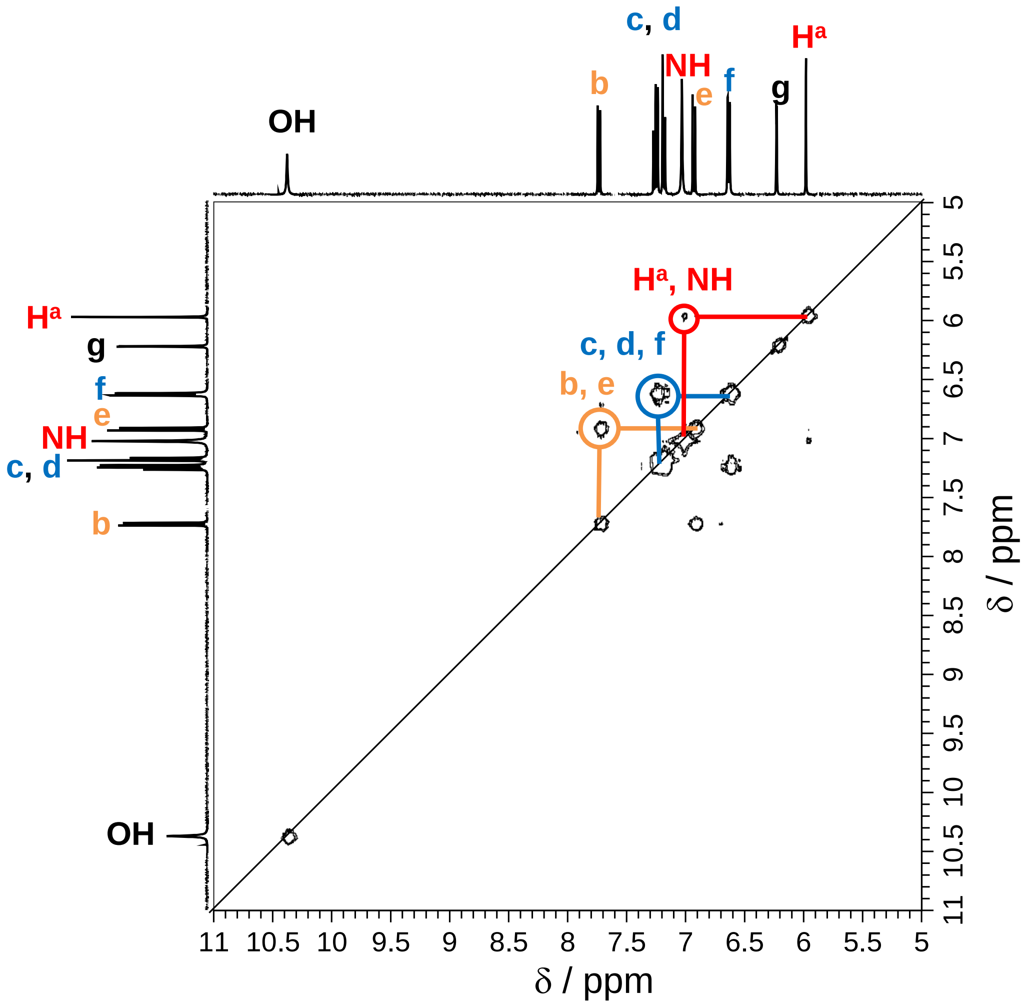

Fig. S8 ^{1}H-^{1}H COSY chart of **1** (30 mM, DMSO–d_6, 400 MHz). Colored circles indicate the observed cross peaks. The texts next to the circle mean the coupling protons.

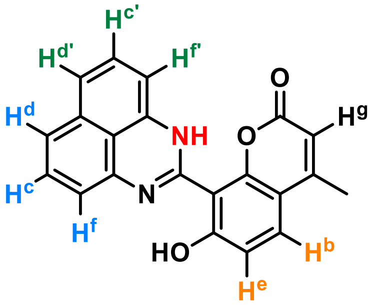

1'

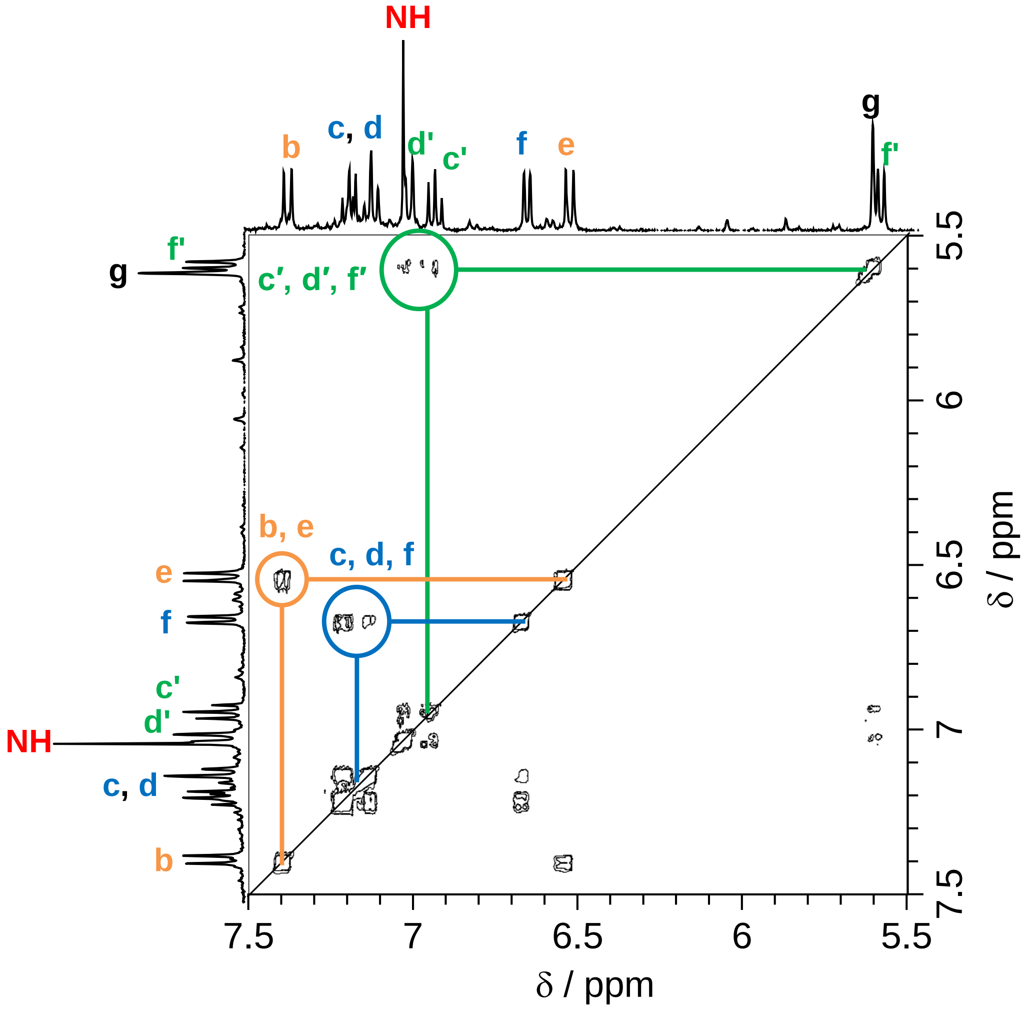

Fig. S9 ^{1}H-^{1}H COSY chart of **1′** (30 mM, DMSO–d$_6$, 400 MHz). Colored circles indicate the observed cross peaks. The texts next to the circle mean the coupling protons.

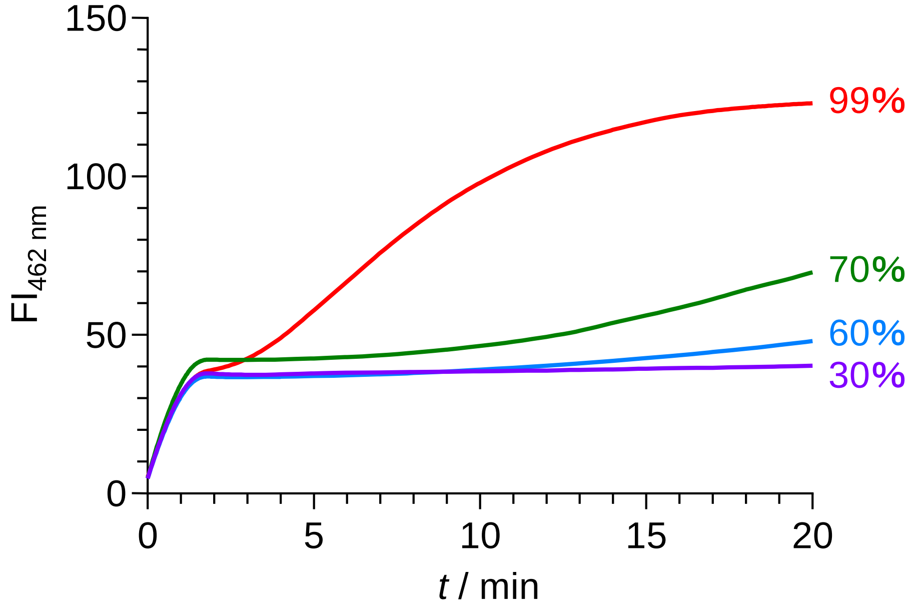

Fig. S10 Time-dependent change in the fluorescence intensity of **1** at 462 nm (10 μM) after addition of OCl^- (50 equiv) measured at 25 °C in MeCN solutions (HEPES 0.1 M, pH 7.0) with different water contents.

Cartesian Coordinates (in Å) of **1** (DFT/B3LYP/6–31+G*)

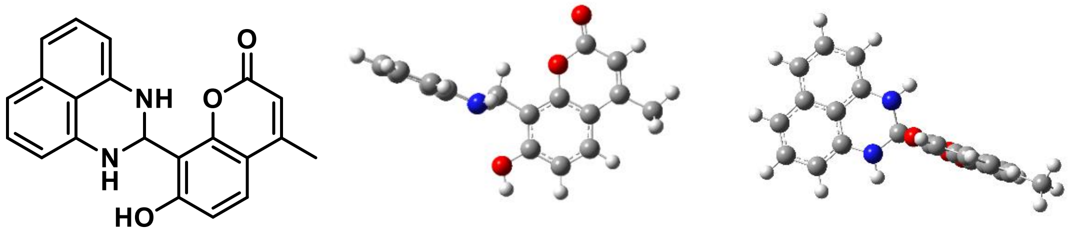

C	-2.59998	2.665419	-0.00016	C	3.059685	-0.05288	-2.43551
C	-1.30104	2.122463	-0.00013	H	-2.72707	3.746045	-0.00022
C	-1.10217	0.727232	-3.1E-05	H	-4.69881	2.275944	-0.00012
C	-2.25129	-0.08475	0.000018	H	-5.22576	-2.56471	0.000175
C	-3.5653	0.436016	-1.2E-05	H	0.169034	-0.99211	0.000113
C	-3.70712	1.835229	-0.0001	H	-0.49296	3.859368	-0.00026
O	-2.04423	-1.43422	0.000097	H	-6.29185	0.645239	0.883376
C	-3.08388	-2.37208	0.00018	H	-6.29183	0.645076	-0.88353
C	-4.4286	-1.82921	0.00013	H	-6.81258	-0.8015	0.000053
C	-4.68196	-0.4931	0.000039	H	0.53197	0.374526	2.045974
C	0.283028	0.10234	0.000012	H	0.531927	0.374141	-2.046
O	-0.21267	2.930196	-0.00019	H	2.539396	0.096748	3.379357
C	-6.09647	0.0244	-1.8E-05	H	4.939409	-0.5332	3.380705
O	-2.7745	-3.54241	0.000175	H	6.165051	-0.8784	1.257772
N	1.043652	0.518103	1.182202	H	6.165027	-0.87863	-1.25769
C	2.379962	0.110716	1.236732	H	4.939344	-0.53382	-3.38066
C	3.066915	-0.08666	0	H	2.539331	0.096131	-3.37938
C	2.379938	0.110493	-1.23675				
N	1.043627	0.517892	-1.18227				
C	3.059732	-0.05244	2.435509				
C	4.426643	-0.40805	2.429927				
C	5.113351	-0.60361	1.248362				
C	4.452032	-0.44108	0.00002				
C	5.113327	-0.60384	-1.24831				
C	4.426596	-0.4085	-2.42989				

Cartesian Coordinates (in Å) of **1′** (DFT/B3LYP/6–31+G*)

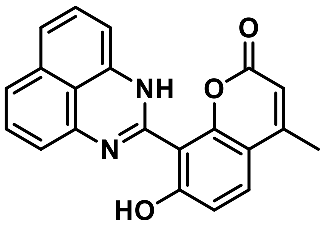

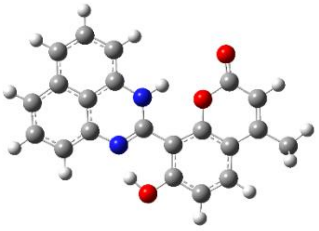
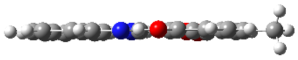

C	-2.39828	2.535728	0.004035	O	0.468111	-3.3021	0.001439
C	-3.77172	2.877929	0.004016	H	-1.63685	3.314675	0.005575
C	-4.76072	1.911836	0.002177	H	-4.04639	3.932516	0.005508
C	-4.41637	0.529281	0.000242	H	-5.81255	2.196413	0.002197
C	-3.03233	0.179967	0.00024	H	-6.43152	-0.28443	-0.00179
C	-2.04167	1.196913	0.002172	H	-5.69292	-2.64723	-0.00507
C	-5.36951	-0.52877	-0.00175	H	-3.26806	-3.23395	-0.00498
C	-4.95165	-1.84821	-0.00358	H	4.800607	-2.23354	0.002606
C	-3.57993	-2.1905	-0.00354	H	2.934406	-3.84995	0.002979
C	-2.62046	-1.18539	-0.00166	H	4.992548	2.628992	-0.00221
N	-1.25928	-1.49611	-0.00159	H	6.247798	-0.50404	0.88488
C	-0.35063	-0.54241	0.000165	H	6.248926	-0.5056	-0.88068
N	-0.7061	0.774861	0.002072	H	6.670849	0.977479	0.00106
C	1.076946	-0.95218	0.000509	H	-0.4405	-2.81595	-9.1E-05
C	2.178517	-0.05788	-0.00022	H	0.02803	1.473898	0.003469
C	3.521757	-0.47823	0.000672				
C	3.776708	-1.86895	0.001968				
C	2.744403	-2.77822	0.002216				
C	1.40121	-2.34592	0.001351				
O	1.90004	1.282537	-0.00205				
C	2.871409	2.285091	-0.00329				
C	4.236283	1.846849	-0.00165				
C	4.569175	0.521925	0.000124				
C	6.01435	0.103494	0.001417				
O	2.454138	3.435226	-0.00571				

Cartesian Coordinates (in Å) of **1″** (DFT/B3LYP/6–31+G*)

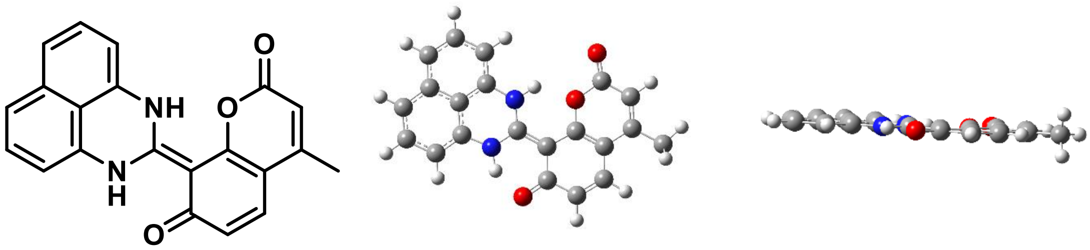

C	-2.37416	2.522559	-0.00129	O	0.483158	-3.3102	-0.00283
C	-3.7451	2.878156	-0.00031	H	-1.60664	3.295546	-0.0021
C	-4.74181	1.92105	0.000792	H	-4.00934	3.935198	-0.00041
C	-4.4108	0.53598	0.000971	H	-5.7911	2.214756	0.001571
C	-3.03094	0.177156	-3.5E-05	H	-6.43313	-0.259	0.002969
C	-2.03019	1.183408	-0.00114	H	-5.71618	-2.62907	0.003345
C	-5.37365	-0.51358	0.002171	H	-3.29944	-3.23985	0.001656
C	-4.96965	-1.83544	0.002371	H	4.815675	-2.22837	0.002315
C	-3.59918	-2.1923	0.001382	H	2.979776	-3.86025	0.000617
C	-2.64666	-1.18944	0.00016	H	4.980906	2.638133	0.002026
N	-1.27325	-1.46935	-0.00077	H	6.249434	-0.48788	0.885605
C	-0.30687	-0.53777	-0.00158	H	6.251229	-0.48776	-0.87957
N	-0.68937	0.758337	-0.00192	H	6.661218	0.997367	0.003554
C	1.084074	-0.97029	-0.0016	H	0.044764	1.459333	-0.00241
C	2.183708	-0.0667	-0.00094	H	-0.89967	-2.44376	-0.00078
C	3.526125	-0.47506	0.000374				
C	3.786633	-1.87659	0.001054				
C	2.77247	-2.79149	0.000165				
C	1.386249	-2.4033	-0.00182				
O	1.897803	1.271479	-0.00137				
C	2.864031	2.286576	-0.00023				
C	4.225091	1.855903	0.001149				
C	4.56151	0.526302	0.001369				
C	6.01094	0.118632	0.00282				
O	2.429016	3.432491	-0.00014				
